# p250GAP Is a Novel Player in the Cdh1-APC/Smurf1 Pathway of Axon Growth Regulation

**DOI:** 10.1371/journal.pone.0050735

**Published:** 2012-11-30

**Authors:** Madhuvanthi Kannan, Shih-Ju Lee, Nicola Schwedhelm-Domeyer, Takanobu Nakazawa, Judith Stegmüller

**Affiliations:** 1 Cellullar and Molecular Neurobiology, Max-Planck-Institute of Experimental Medicine, Göttingen, Germany; 2 Department of Neurophysiology, School of Medicine, University of Tokyo, Tokyo, Japan; Harvard University, United States of America

## Abstract

Axon growth is an essential process during brain development. The E3 ubiquitin ligase Cdh1-APC has emerged as a critical regulator of intrinsic axon growth control. Here, we identified the RhoGAP p250GAP as a novel interactor of the E3 ubiquitin ligase Cdh1-APC and found that p250GAP promotes axon growth downstream of Cdh1-APC. We also report that p250GAP undergoes non-proteolytic ubiquitination and associates with the Cdh1 substrate Smurf1 to synergistically regulate axon growth. Finally, we found that *in vivo* knockdown of p250GAP in the developing cerebellar cortex results in impaired migration and axonal growth. Taken together, our data indicate that Cdh1-APC together with the RhoA regulators p250GAP and Smurf1 controls axon growth in the mammalian brain.

## Introduction

Axon growth is a fundamental process in the developing nervous system [1]–[3]. The ubiquitin proteasome system (UPS) has emerged as a key player in neuronal development [4], [5]. A growing number of E3 ubiquitin ligases have been implicated in axon growth and guidance, in addition to other crucial neurodevelopmental events including neurogenesis, polarity, migration, dendritic growth, synapse formation, and memory [6]–[16].

The multisubunit E3 ubiquitin ligase Cdh1-APC, a crucial cell cycle regulator in mitotic cells, acts as a suppressor of axon growth in neurons [12]. Several studies have delineated an intricate mechanism by which Cdh1-APC exerts a strong intrinsic inhibition of axon growth [17]–[21]. Like in mitotic cells, Cdh1-APC targets several substrates for proteasomal degradation in neurons to control axon growth suppression. The transcriptional regulators SnoN and Id2 and the E3 ligase Smurf1 have been identified as bona fide, signature D-box motif-harboring substrates [16], [19], [22]. While SnoN and Id2 are nuclear targets of Cdh1 in axon growth control [17], [19], Smurf1 acts both in the nucleus and cytoplasm to stimulate axon growth [16]. Importantly, cytoplasmic Smurf1 targets the small GTPase RhoA for degradation [6], [23]. RhoA, which is instrumental to axon formation, axon growth and guidance, and neuronal migration [24], [25], relays myelin-induced axon growth inhibition [26], [27] and has emerged as a crucial intrinsic suppressor of axon growth downstream of the Cdh1-APC/Smurf1 pathway [16]. Although Cdh1-APC’s activity is crucial in the nucleus to control axon growth [17], regulation of RhoA at the axonal growth cone arose as an important aspect in Cdh1-APC-mediated axon growth inhibition. Kannan and colleagues recently identified a novel pathway, in which Cdh1-APC acts upstream of the E3 ligase for RhoA, Smurf1 [16].

While Cdh1-APC targets the RhoA-E3 ligase Smurf1 for degradation, we reasoned that Cdh1 may also interact with and control regulators of RhoA activity owing to the opposing effect of constitutively active RhoA on Cdh1 knockdown-enhanced axon growth [16]. The activity of Rho GTPases switches from an active to an inactive state and vice versa, which is tightly controlled by specific binding of GAPs (GTPase activating proteins) and GEFs (guanine nucleotide exchange factors), respectively. Hence we carried out a RhoGAP interaction screen and identified the RhoGAP p250GAP as a novel interactor of the E3 ubiquitin ligase Cdh1-APC. p250GAP promotes axon growth downstream of Cdh1-APC and in a synergistic manner with Smurf1. Finally, we demonstrate that p250GAP and Smurf1 promote axon growth and migration in the developing mammalian nervous system.

## Results

### The RhoGAP p250GAP is a Novel Interactor of Cdh1

**Figure 1:**
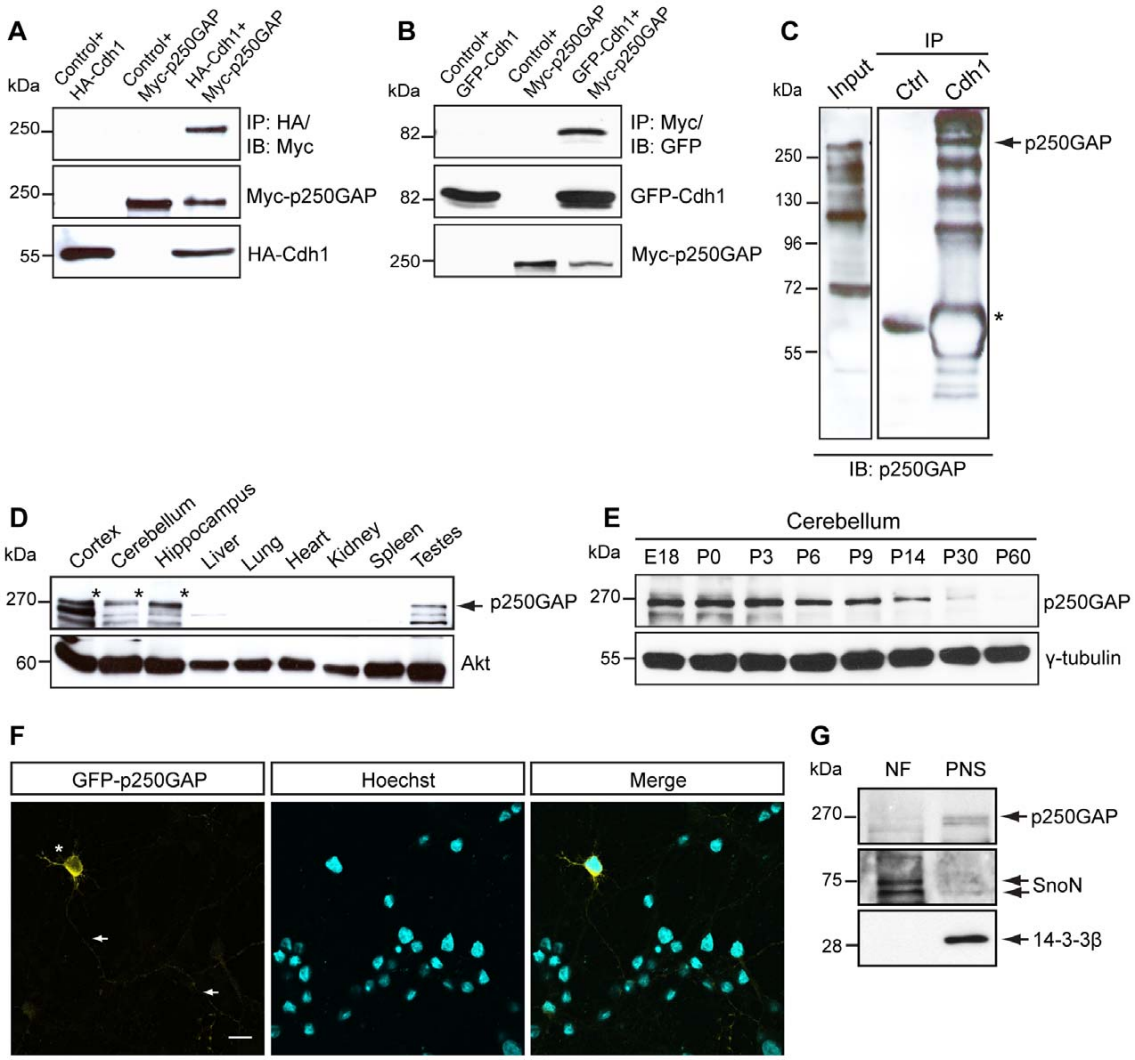
Brain-dominant p250GAP associates with Cdh1. A. 293T cells transfected with control vector pcDNA3 and HA-Cdh1, or control pCMV5 vector and Myc-p250GAP, or both HA-Cdh1 and Myc-p250GAP plasmids, were lysed and lysates subjected to immunoprecipitation with the HA antibody and immunoblotting with the Myc antibody. **B.** Lysates of 293T cells transfected with control vector pcDNA3 and GFP-Cdh1, or GFP and Myc-p250GAP plasmid, or plasmids encoding GFP-Cdh1 and Myc-p250GAP were subjected to immunoprecipitation with the Myc antibody followed by immunoblotting with the GFP antibody. **C.** Total brain lysate from P9 mouse was subjected to immunoprecipitation with control serum or Cdh1 antiserum followed by immunoblotting with p250GAP antiserum. Arrow indicates p250GAP and asterisk IgG_H_. **D.** Lysates of indicated tissues were collected from wild type P60 mouse and subjected to immunoblotting using the p250GAP antibody. Akt served as loading control. Asterisks indicate p250GAP. **E.** Cerebella were collected from wild type rats at the indicated days and the lysates were subjected to immunoblotting using the p250GAP antibody. γ-tubulin served as loading control. E = embryonic, P = postnatal. **F.** Granule neurons were transfected at DIV 1 with GFP-p250GAP and at DIV 3 subjected to immunocytochemistry with the GFP antibody and the nuclear dye bisbenzimide Hoechst 33258. Arrows and asterisk indicate axons and soma, respectively. Scale bar equals 10 µm. **G.** Cerebellar granule neurons were subjected to subcellular fractionation at DIV 3. Nuclear (NF) and postnuclear supernatant (PNS) fractions were immunoblotted using p250GAP, SnoN and 14-3-3β antibodies. SnoN and 14-3-3β served as controls for NF and PNS, respectively.

We have previously characterized a novel Cdh1-APC/Smurf1 pathway of axon growth, in which RhoA emerged as a crucial downstream component of intrinsic axon growth suppression [16]. This prompted us to investigate other regulators of RhoA and more specifically their interaction with Cdh1. Since both Cdh1-APC and RhoA negatively regulate axon growth, we hypothesized that a Rho GTPase activating protein (GAP) would meet the requirement to establish a connection between Cdh1-APC and RhoA. We thus selected brain-abundant GAPs, which were previously implicated in neuronal morphogenesis, and screened those for an association with Cdh1. In coimmunoprecipitation experiments, we analyzed the association of Cdh1 with the RhoGAPs Nadrin, p190GAP, GRAF, oligophrenin and p250GAP in heterologous cells. Among these GAPs, we found a robust association of Cdh1 and p250GAP **(** **Figure 1A** **, Figure S1A–E).** In reciprocal analysis, we immunoprecipitated p250GAP and found that Cdh1 interacts with p250GAP (**Figure 1B**). In addition, we found that Cdh1 and p250GAP associate endogenously in the brain (**Figure 1C**). These data identify the RhoGAP p250GAP as a novel interactor of Cdh1.

**Figure 2:**
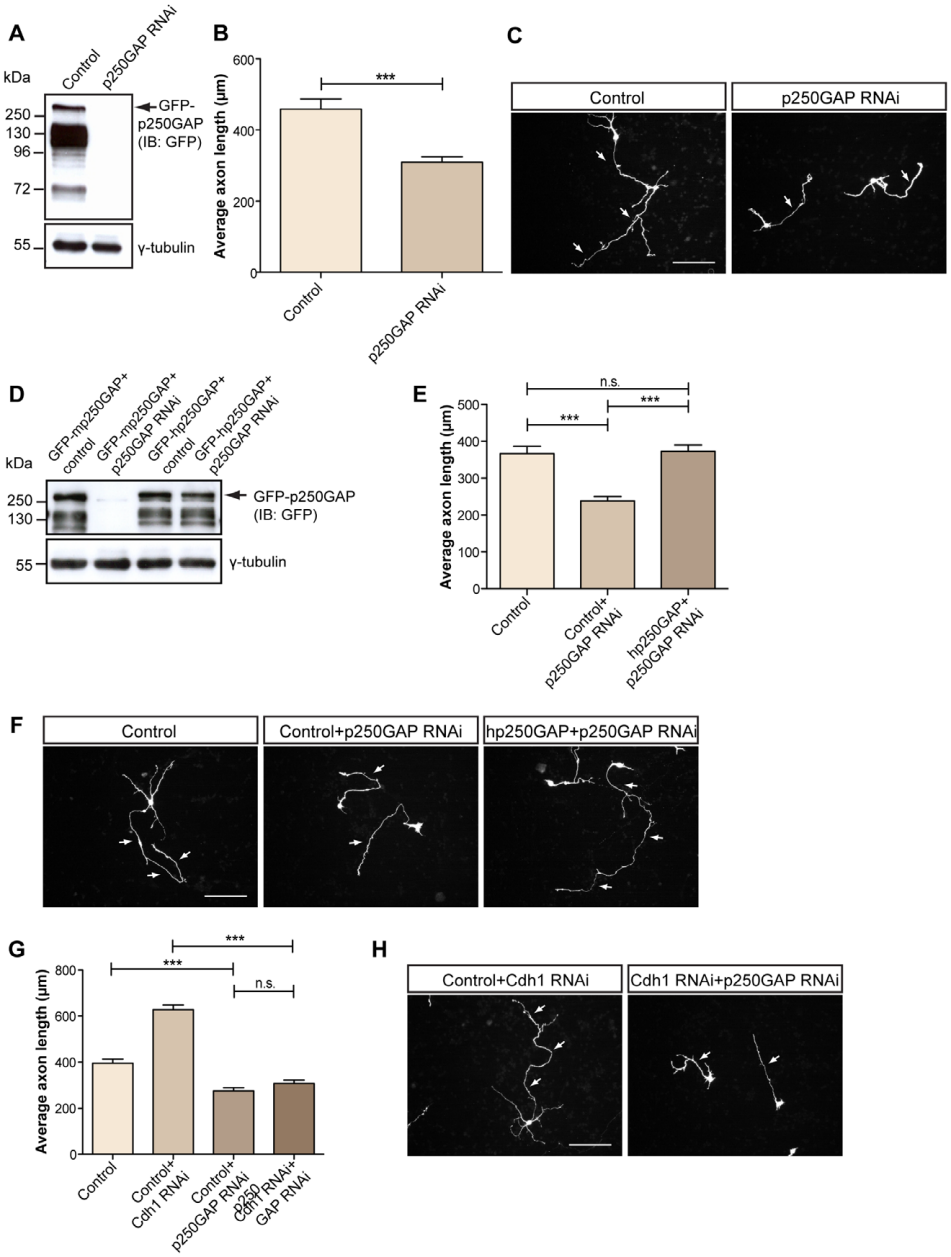
p250GAP promotes axon growth. A. Lysates of 293T cells transfected with GFP-p250GAP plasmid together with control vector U6 or the p250GAP RNAi plasmid were subjected to immunoblotting using the GFP antibody. γ-tubulin served as the loading control. **B.** Cerebellar granule neurons were transfected at DIV 0 with control vector U6 or the p250GAP RNAi plasmid together with GFP and BCL_XL_ plasmids and maintained in conditioned media. At DIV 4, neurons were subjected to immunocytochemistry using the GFP antibody. Axonal length was measured in GFP-positive transfected neurons using ImageJ software. A total of 300 neurons were measured (t-test, ***p<0.0001, values indicate mean+SEM). **C.** Representative images of transfected neurons in **B.** Scale bar equals 100 µm. Arrows indicate axons. **D.** Lysates of 293T cells transfected with GFP-tagged mouse (m)p250GAP or human (h)p250GAP together with control vector U6 or rodent-specific p250GAP RNAi plasmid were immunoblotted with the GFP antibody. γ-tubulin served as the loading control. **E.** Granule neurons were transfected at DIV 0 with control vectors pcDNA3 and U6, or p250GAP RNAi together with pcDNA3 or human p250GAP plasmids. At DIV 4, neurons were analyzed as in **2B**. A total of 273 neurons were measured (t-test, ***p<0.0001, n.s. = not significant, values indicate mean+SEM). **F.** Representative images of transfected neurons in **E.** Scale bar equals 100 µm. Arrows indicate axons. **G.** Granule neurons were transfected at DIV 0 with control U6, U6 and Cdh1 RNAi, or U6 and p250GAP RNAi or both Cdh1 RNAi and p250GAP RNAi plasmids and were subjected to axon growth assays at DIV4 as described in **2B**. A total of 635 neurons were measured (ANOVA, ***p<0.0001, n.s. = not significant, values indicate mean+SEM). **H.** Representative images of transfected neurons in **G**. Scale bar equals 100 µm. Arrows indicate axons.

**Figure 3:**
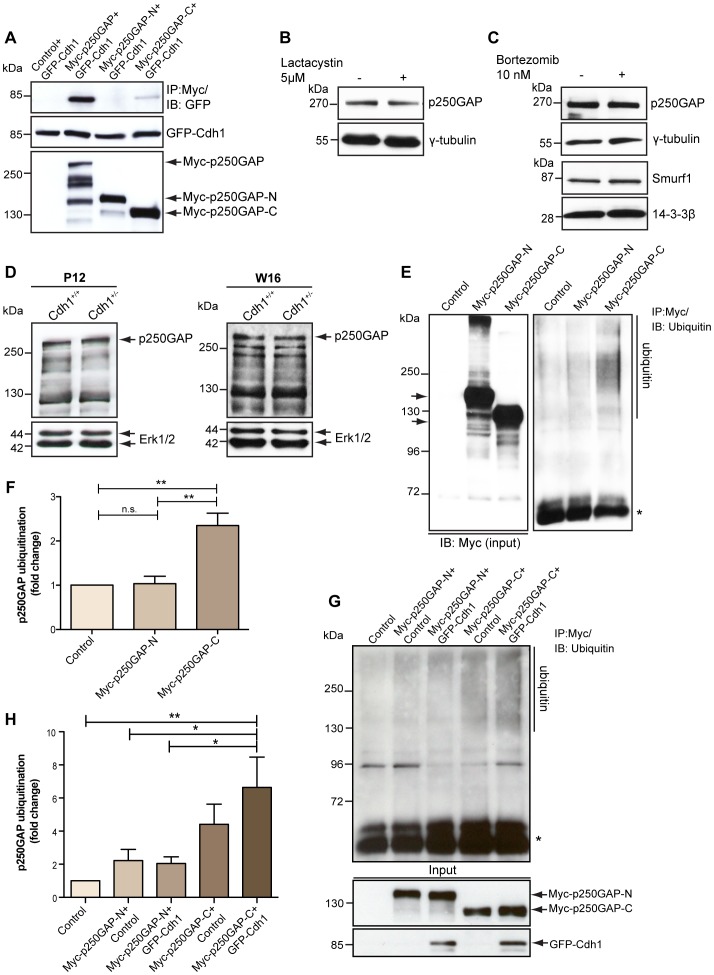
p250GAP is ubiquitinated but not degraded by the proteasome. A. 293T cells were transfected with GFP-Cdh1 together with control vector pCMV-Myc, or plasmids encoding Myc-tagged full length, N- or C-terminal fragments of p250GAP (Myc-p250GAP, Myc-p250GAP-N or Myc-p250GAP-C) and the lysates were subjected to immunoprecipitation with the Myc antibody followed by immunoblotting with the GFP antibody. **B.** Granule neurons treated with vehicle or 5 µM lactacystin for 10 h following which lysates were subjected to immunoblotting using the p250GAP antibody. γ-tubulin served as the loading control. **C.** Lysates of granule neurons treated with vehicle or 10 nM bortezomib for 10 h were subjected to immunoblotting using the p250GAP or Smurf1 antibodies. γ-tubulin and 14-3-3β served as loading controls, respectively. **D.** Cerebellar lysates of postnatal day (P) 12 and week (W) 16 Cdh1^+/+^ and Cdh1^+/−^ mice were immunoblotted using the p250GAP antibody. Erk1/2 served as loading control. **E.** 293T cells were transfected with control vector pCMVmyc, Myc-p250GAP-N or Myc-p250GAP-C plasmid and the lysates were immunoprecipitated using the Myc antibody followed by immunoblotting with ubiquitin antibody. Asterisk indicates IgG_H_. **F.** Intensity of p250GAP ubiquitination in each condition in **B** was quantified and normalized to that of control using ImageJ software (ANOVA, **p<0.01, n.s. = not significant, values indicate mean+SEM). **G.** 293T cells were transfected with control vectors pCMV-Myc and pEGFP, Myc-p250GAP-N plasmid together with pEGFP or GFP-Cdh1 plasmid, or Myc-p250GAP-C plasmid together with pEGFP or GFP-Cdh1 plasmid and the lysates were immunoprecipitated using the Myc antibody followed by immunoblotting with ubiquitin antibody. Asterisk indicates IgG_H_. **H.** Intensity of p250GAP ubiquitination in each condition in **D** was quantified and normalized to that of control using ImageJ software (ANOVA, *p<0.05, **p<0.01, values indicate mean+SEM).

**Figure 4:**
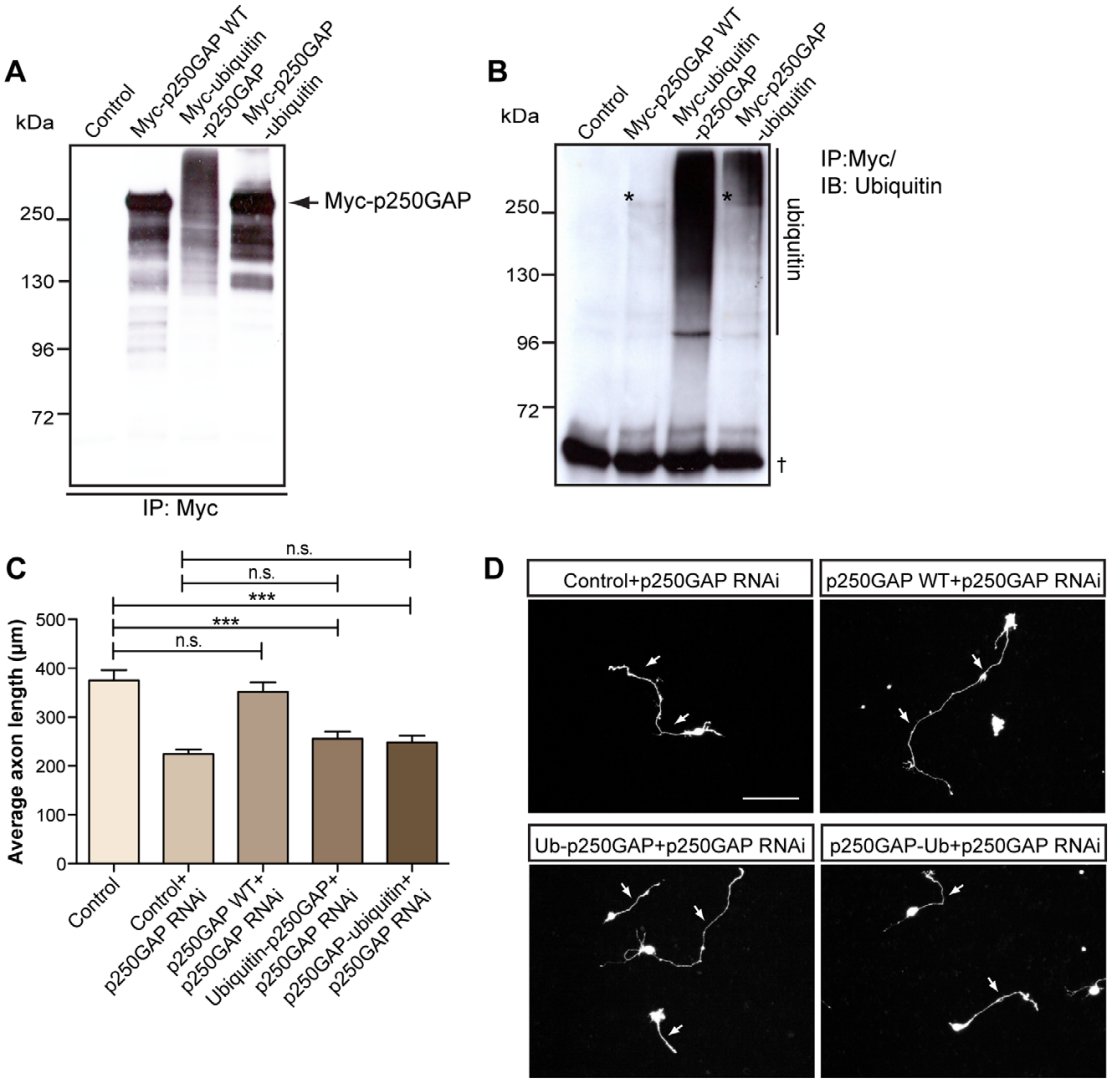
p250GAP-ubiquitin displays loss-of-function in axon growth. A. 293T cells were transfected with pcDNA3 control vector, Myc-p250GAP wild type (Myc-p250GAP WT), Myc-ubiquitin-p250GAP or Myc-p250GAP-ubiquitin plasmid and lysates were subjected to immunoblotting using the Myc antibody. **B.** Lysates of 293T transfected with pcDNA3 control vector, Myc-p250GAP WT, Myc-ubiquitin-p250GAP or Myc-p250GAP-ubiquitin plasmid were subjected to immunoprecipitation using the Myc antibody followed by immunoblotting with ubiquitin antibody. Asterisks indicate ubiquitinated p250GAP and † indicates IgG_H_. **C.** Granule neurons were transfected at DIV 0 with the control vector pcDNA3 together with U6 or the p250GAP RNAi plasmid, or p250GAP RNAi together with p250GAP WT, Ubiquitin-p250GAP or p250GAP-ubiquitin plasmids. At DIV 4, neurons were subjected to axon assays as described in **2B**. A total of 533 neurons were measured (ANOVA, ***p<0.0001, n.s. = not significant, values indicate mean+SEM). **D.** Representative images of transfected neurons in **C.** Scale bar equals 100 µm. Arrows indicate axons.

**Figure 5:**
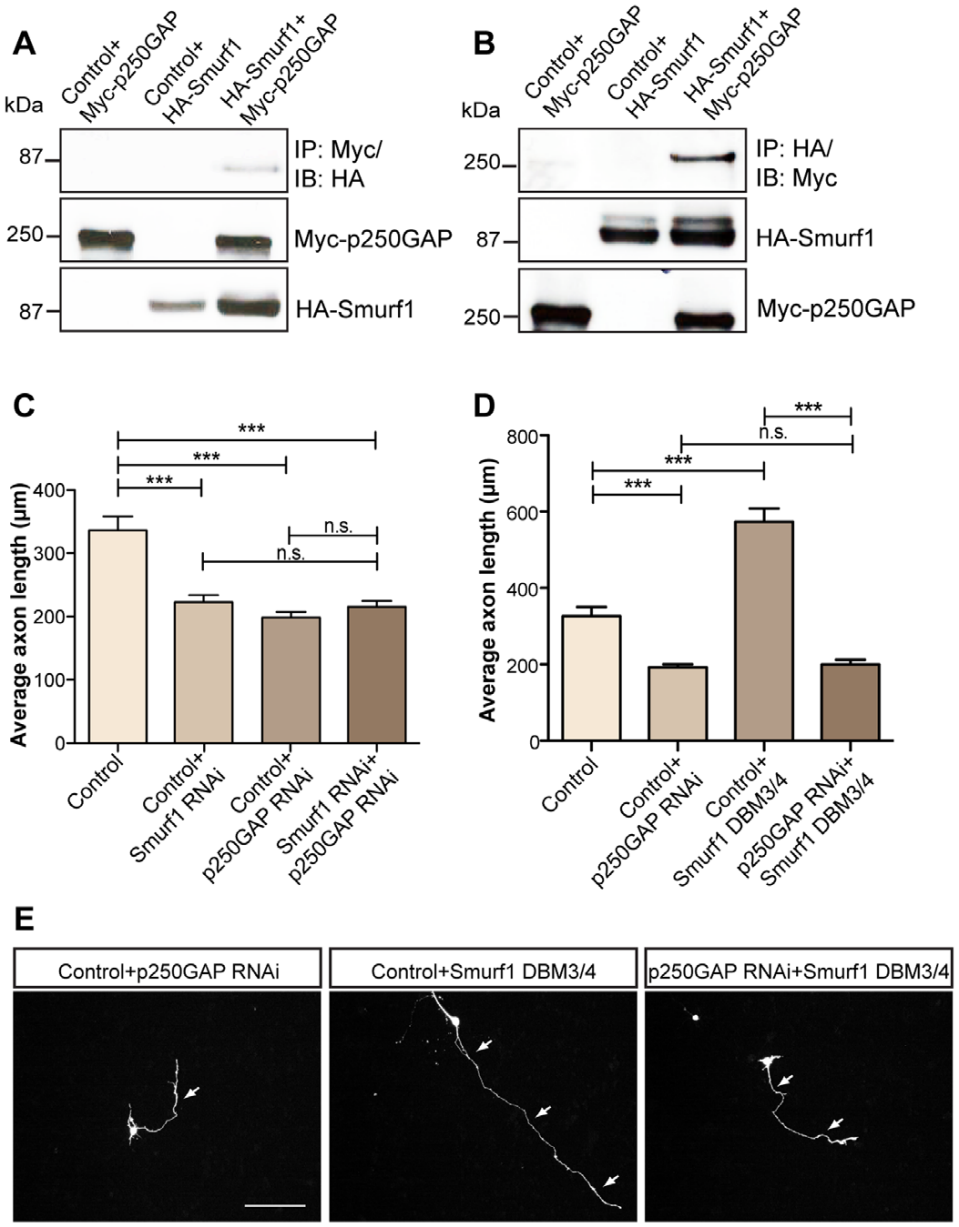
Association of p250GAP with Smurf1 regulates axon growth. A. 293T cells were transfected with control vector pCMV5 and Myc-p250GAP, or control pcDNA3 vector and HA-Smurf1, or both HA-Smurf1 and Myc-p250GAP plasmids and the lysates were subjected to immunoprecipitation with the Myc antibody followed by immunoblotting with the HA antibody. **B.** Reciprocal co-immunoprecipitation of **A**. **C.** Granule neurons transfected with control U6, U6 and Smurf1 RNAi, or U6 and p250GAP RNAi or both Smurf1 RNAi and p250GAP RNAi plasmids were analyzed at DIV 4 as described in **2B.** A total of 467 neurons were measured (ANOVA, ***p<0.0001, n.s. = not significant, values indicate mean+SEM). **D.** Granule neurons transfected with control U6, pCMV5 and p250GAP RNAi, or U6 and Smurf1 DBM3/4 or both p250GAP RNAi and Smurf1 DBM3/4 plasmids were subjected to axon growth assays at DIV 4 as described in **2B.** A total of 324 neurons were measured (ANOVA, ***p<0.0001, n.s. = not significant, values indicate mean+SEM). **E.** Representative images of transfected neurons in **D.** Scale bar equals 100 µm. Arrows indicate axons.

**Figure 6:**
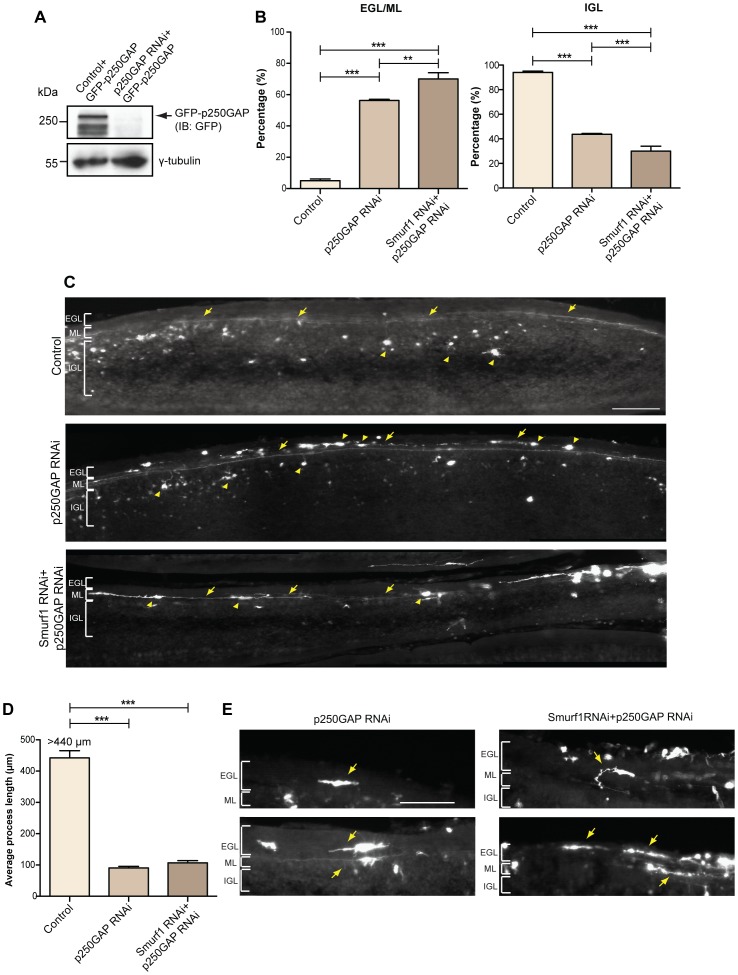
p250GAP promotes migration and axon extension in the developing cerebellar cortex. A. Lysates of 293T cells transfected with control vector U6-CMV-EGFP or U6-p250GAP RNAi-CMV-EGFP (p250GAP RNAi) together with GFP-p250GAP expression plasmid were subjected to immunoblotting with GFP and γ-tubulin (loading control) antibodies. **B.** The control U6-CMV-EGFP plasmid, p250GAP RNAi-CMV-EGFP (p250GAP RNAi), or p250GAP RNAi and Smurf1 RNAi-CMV-EGFP (Smurf1 RNAi) plasmids together with BCL_XL_ plasmid were injected into the cerebellum of P4 rat pups. At P9, cerebella were isolated and coronal sections were subjected to immunohistochemistry using the GFP antibody. The location of transfected neurons was assessed as either external granule layer (EGL)/molecular layer (ML) or internal granule layer (IGL). A total of 10527 neurons were counted (ANOVA, **p<0.01, ***p<0.0001, values indicates mean+SEM). **C.** Representative images of each condition in **B.** Scale bar equals 100 µm. Arrows indicate parallel fibers and processes and arrowheads indicate cell bodies. **D.** Quantification of axonal length of neurons in the control, p250GAP knockdown and p250GAP/Smurf1 double knockdown conditions. A total of 325 neurons were measured (ANOVA, ***p<0.0001, values indicate mean+SEM). **E.** Representative images of the knockdown conditions in **C.** EGL = external granule layer, ML = molecular layer, IGL = internal granule layer. Scale bar equals 100 µm. Arrows indicate parallel fibers and processes.

**Figure 7:**
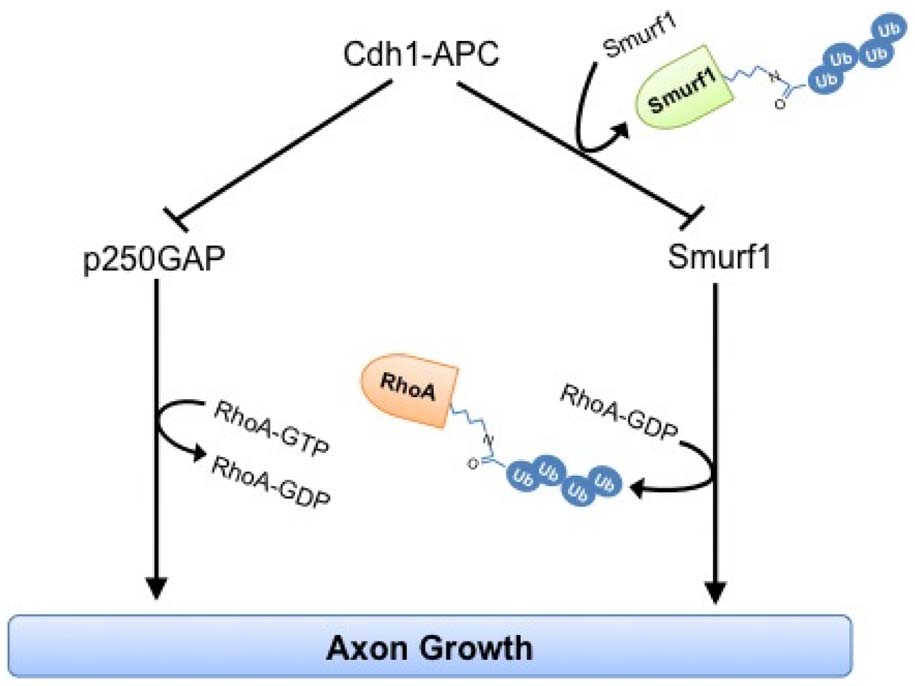
Schematic of Cdh1-APC-regulated axon growth. The E3 ubiquitin ligase Cdh1-APC operates upstream of the RhoA-regulating proteins Smurf1 and p250GAP in the control of axon growth. While Cdh1-APC ubiquitinates Smurf1 for proteasomal degradation, it ubiquitinates p250GAP for a functional modification in RhoA-dependent axon growth inhibition.

p250GAP is highly enriched in the brain (**Figure 1D**), [28] and has been identified as a GAP, which regulates both RhoA and Cdc42 activity in neurons [28], [29]. To examine the temporal expression of p250GAP in the cerebellum, we isolated rat cerebella at different ages and immunoblotted lysates with the p250GAP antibody and found that while p250GAP is abundantly expressed in early developmental stages, its levels decrease over time (**Figure 1E**). At the subcellular level, expression of GFP-p250GAP demonstrates a cytoplasmic localization of p250GAP in granule neurons (**Figure 1F**). This finding was supported by subcellular fractionation analyses of granule neuron lysates revealing p250GAP as a cytoplasmic protein (**Figure 1G**). Taken together these experiments characterize p250GAP as a brain-enriched, cytoplasmic protein.

### p250GAP Promotes Axon Growth

Owing to p250GAP’s neuronal expression and interaction with Cdh1, we took an RNAi approach to ask whether p250GAP plays a role in axon growth regulation. Nakazawa and colleagues previously identified a targeting region in p250GAP [29], which we utilized to generate a plasmid encoding a p250GAP hairpin. We confirmed the efficient knockdown of GFP-tagged p250GAP in heterologous cells (**Figure 2A**) prior to transfection of granule neurons with the control plasmid or the p250GAP RNAi plasmid. Axon growth measurements of p250GAP knockdown neurons uncovered a significant reduction in axonal length as compared to control neurons (**Figure** **2B, C**). To determine that this phenotype can be specifically attributed to knockdown of p250GAP and to rule out off-target effects, we validated an RNAi-resistant form of p250GAP (human (h)p250GAP) with immunoblotting (**Figure 2D** **)**. Then we carried out rescue experiments and coexpressed the RNAi-resistant form of p250GAP in the background of p250GAP knockdown and found that axonal length is restored to baseline length of control neurons (**Figure 2E, F** **)**.

To establish a Cdh1/p250GAP pathway of axon growth control, we carried out epistasis analyses. We found that while Cdh1 knockdown promotes and p250GAP RNAi inhibits axon growth, the p250GAP RNAi phenotype overrules the enhancement of axon growth triggered by Cdh1 knockdown (**Figure 2G, H**), indicating that p250GAP acts downstream of Cdh1-APC.

### p250GAP is Ubiquitinated but not Degraded by the Proteasome

To investigate if p250GAP is a substrate of Cdh1-APC, we screened the p250GAP amino acid sequence for Cdh1 signature binding motifs including D-box and KEN box. We found only one potential D-box in the N-terminal half of p250GAP and reasoned that since Cdh1 binds to the C-terminal part of p250GAP (**Figure 3A**), this D-box is unlikely to be a bona fide one. Also, mutation of the D-box does not alter the binding affinity of p250GAP to Cdh1 (**Figure S2**). We then tested whether neuronal p250GAP is sensitive to proteasome inhibition, which indicates a proteasome-dependent turnover. We treated granule neurons with the proteasome inhibitors lactacystin, bortezomib or with vehicle, analyzed equal amounts of lysates with immunoblotting using the p250GAP antibody and found that p250GAP fails to respond to either inhibitor (**Figure 3B, C**), while Smurf1 responds to bortezomib (**Figure 3C**) and as shown previously to lactacystin [16]. To further underscore that p250GAP may not be a substrate of Cdh1-APC targeted for degradation, we analyzed p250GAP levels in wild type and heterozygous Cdh1 gene trap animals. Homozygous Cdh1 gene trap animals are not viable owing to Cdh1’s essential function in cell cycle [30]. While we previously found an accumulation of the Cdh1 target Smurf1 [16], p250GAP levels are unaltered in lysates of P12 and W16 cerebellum of heterozygous animals as compared to wild type littermates (**Figure 3D**). These data indicate that p250GAP is not targeted for proteasomal degradation by Cdh1-APC.

To examine whether p250GAP might undergo non-proteolytic and possibly activity-modifying ubiquitination, we first determined in cell-based ubiquitination assays whether the p250GAP-N and p250GAP-C deletion mutants are modified by ubiquitin. We found in immunoprecipitation analysis that p250GAP-C but not the p250GAP-N presents with a ubiquitin smear, suggesting the modification by ubiquitin at the C-terminus (**Figure 3E, F**). In cell-based ubiquitination assays, we found that expression of Cdh1 selectively enhances the ubiquitination of p250GAP-C but not p150GAP-N (**Figure 3G, H**). These results suggest that Cdh1 stimulates the ubiquitination of p250GAP.

### A p250GAP-ubiquitin Fusion Protein is Rendered Non-functional in Axon Growth Stimulation

Functional modification of proteins by ubiquitination including monoubiquitination has been demonstrated for other proteins e.g. the GTPase Rap2a, which undergoes mono- or diubiquitination by the E3 HECT ligase NEDD4 in the control of neurite development [31]. To test the idea of functional modification of p250GAP by ubiquitination, we constructed two p250GAP fusion proteins, one that harbors an N-terminal ubiquitin and one that harbors ubiquitin at the C-terminus. Previous experiments have established that an N-terminal ubiquitin moiety subjects a protein to the N-end rule of protein degradation by the proteasome, while a C-terminally appended ubiquitin results in stabilization [32], [33]. Our experiments revealed that the ubiquitin-p250GAP fusion protein is highly unstable. p250GAP-ubiquitin however is as stable as the wild type p250GAP (**Figure 4A** **)**. We subsequently immunoprecipitated p250GAP WT, ubiquitin-p250GAP or p250GAP-ubiquitin, followed by immunoblotting using the ubiquitin antibody and found that while N-terminal ubiquitination of p250GAP produces a degradation smear, p250GAP-ubiquitin displays a high molecular weight smear above 250kD and wild type p250GAP exhibits a lesser extent of ubiquitin reactivity (**Figure 4B**). To determine whether appending ubiquitin to p250GAP results in a change of function, we carried out a rescue analysis in an analogous experiment as described in **Figure 2E**. We expressed wild type p250GAP, ubiquitin-p250GAP or p250GAP-ubiquitin in the background of p250GAP RNAi and found again restoration of axon growth by wild type p250GAP. Expectedly, ubiquitin-p250GAP, which is prone to degradation, results in loss-of-function (**Figure 4C**). Interestingly, the stable version of p250GAP, p250GAP-ubiquitin falls short to rescue axon growth (**Figure 4C** **and** **Figure 4D**). Our data support the notion that by undergoing C-terminal ubiquitination, p250GAP is stable but fails to promote axon growth.

### Epistasis Analyses of p250GAP- and Smurf1-regulated Axon Growth

To establish a relationship between Cdh1-APC, p250GAP and the previously identified substrate Smurf1 [16], we went on and tested whether Smurf1 and p250GAP may interact, since both proteins are regulators of RhoA [23], [28]. Strikingly, we found that Smurf1 associates with p250GAP (**Figure 5A, B**). We then examined whether Smurf1 and p250GAP synergistically control axon growth and triggered p250GAP/Smurf1 double knockdown and compared axon length to p250GAP or Smurf1 knockdown or control-transfected neurons. We found that p250GAP double knockdown does not result in significantly shorter axons as compared to single knockdown neurons, suggesting a synergistic effect of p250GAP and Smurf1 in axon growth control (**Figure 5C**). We also investigated which of the two proteins exerts the dominant phenotype. Interestingly, we found that knockdown of p250GAP nullifies the enhancement of axon growth by Smurf1 DBM3/4 (**Figure 5D, E**). These experiments indicate that Smurf1 and p250GAP synergistically regulate axon growth downstream of Cdh1-APC.

### Smurf1 and p250GAP Control Axon Growth and Migration in the Developing Cerebellum

Finally, we explored the role of p250GAP in axonal development in the cerebellar cortex using the *in vivo* electroporation techniques [12], [17]. We injected bicistronic plasmids encoding p250GAP targeting hairpins and an EGFP expression cassette, which were validated for efficient knockdown (**Figure 6A** **)**, into the cerebellum of postnatal day 4 rat pups and subjected the animals to repeated electric pulses. Five days later, we analyzed coronal sections of control and knockdown animals. In control animals, the GFP-positive granule neurons descend into the internal granule layer, form bifurcated axons and develop a dendritic arbor. We have previously shown that Smurf1 knockdown results in axon growth and migration defect [16]. Here, we discovered that both knockdown of p250GAP and p250GAP/Smurf1 double knockdown display an overt phenotype, in which granule neurons are stalled in the molecular layer. When we quantified the migration defect, we found that more than 90% of control neurons migrate into the internal granule layer, while only 43% of p250GAP knockdown neurons descend (**Figure 6B, C**). The double knockdown revealed an even more dramatic failure of neurons to migrate. Here, only 30% reach the internal granule layer, possibly as consequence of additive effects of p250GAP- and Smurf1-regulated migration (**Figure 6B**). In addition, we observed that stalled neurons extend shorter and rather thick axons, which fail to develop into the parallel fibers. The quantification of thick and thin processes demonstrated a clear defect in p250GAP as well as the p250GAP/Smurf1 double knockdown neurons, suggesting that p250GAP and Smurf1 are required for the proper development of parallel fibers (**Figure S3**). Furthermore, we determined axon length of the transfected neurons. Since it is impossible to measure the entire length of parallel fibers of control neurons, we determined the minimal length of these processes by measuring the length of the parallel fibers for as long as we could trace them in the given sections. In the knockdown conditions however, we could determine the tips of the short grown axons and measure their lengths. We found that control axons extend for at least 440 µm, while knockdown of p250GAP or double knockdown results in an average length of 90 µm and 106 µm, respectively (**Figure 6D, E**). Taken together, the *in vivo* analyses demonstrate that p250GAP and Smurf1 promote proper migration and axon elongation in the developing cerebellar cortex.

## Discussion

In this study, we provide further insight into Cdh1-APC controlled axon growth in the mammalian brain. We identified the RhoGAP p250GAP as a novel interactor of Cdh1, that acts downstream of Cdh1 in axon growth regulation. Our data also suggest an interaction and a synergistic action of p250GAP and Smurf1. Finally, we report an axon growth- and migration-stimulating role of p250GAP in the developing cerebellar cortex.

Our previous finding that regulation of the small GTPase RhoA by Smurf1 is an important event in the Cdh1-APC pathway of axon growth suppression [16], led us to discover the GTPase activating protein (GAP) p250GAP as a novel interactor of Cdh1. p250GAP is a large, brain-enriched GAP, which hydrolyses RhoA-GTP and Cdc42-GTP and was found to regulate neurite growth in a neuroblastoma and PC12 cell line [28], [29], [34]. In mature neurons, p250GAP also localizes to dendritic spines to regulate their morphology in an NMDA-receptor mediated manner [29]. Our study revealed that p250GAP operates downstream of Cdh1-APC to promote axon growth. The finding that Smurf1 and p250GAP act synergistically in axon growth indicates that the regulation of RhoA by protein turnover and GAP-dependent inactivation is required for axon growth stimulation. The role of p250GAP in neurite growth was investigated in another study, which employed mouse genetics to examine RICS (p250GAP) in neurons isolated from knockout animal. Here, the authors proposed that RICS (p250GAP) inhibits neurite growth and that it acts as a GAP for Cdc42 and Rac1 but not RhoA [35]. This is however inconsistent with our study and with the findings of Nakazawa and colleagues, which propose that p250GAP enhances axon growth and that, p250GAP acts on RhoA, respectively.

Our study does not support the notion that p250GAP undergoes degradation-initiating ubiquitination as p250GAP protein levels neither respond to proteasome inhibition nor to half of the *Cdh1* gene dosage in the cerebellum. It is thus likely that p250GAP’s ubiquitin modification alters its function. Here, one could envision two plausible scenarios of how ubiquitinated p250GAP fails to promote axon growth: Ubiquitination may render p250GAP inactive as a RhoGAP, which is then unable to inactivate RhoA, or ubiquitinated p250GAP may no longer be able to bind to RhoA and consequently fails to act as a RhoGAP. Non-proteolytic ubiquitination resulting in inactivation or activation has been demonstrated for example in transcriptional regulation [36]. In the brain, Kawabe and colleagues discovered that mono- or diubiquitination of small GTPase Rap2a in neurons triggers inactivation followed by inhibition of dendritic growth [31]. We found that a p250GAP-ubiquitin fusion protein exhibits a loss-of-function to promote axon growth, supporting a potential inactivation as a consequence of Cdh1-mediated ubiquitination.

Interestingly, not only did we find that p250GAP and Smurf1 promote axon growth [16], we also identified the two as novel interactors. This finding raises the question if this interaction is dependent on RhoA. It is conceivable that RhoA mediates the binding of Smurf1 and p250GAP, in particular given that Smurf1 preferentially binds to inactive or nucleotide-free RhoA [37] and that the nature of p250GAP is to inactivate RhoA [28]. It is also possible that owing to the large size of p250GAP, it acts as a platform to recruit Smurf1 and to facilitate Smurf1’s access and binding to RhoA.

In addition to axon growth, we found that p250GAP promotes the proper migration of granule neurons in the cerebellum. The RICS (p250GAP) knockout study however did not examine *in vivo* developmental defects [35]. Further analyses of these mice might be informative and important owing to the role of p250GAP in spine development and migration and possible cognitive defects resulting thereof. Our *in vivo* analyses demonstrate that p250GAP/Smurf1 double knockdown leads to a severe defect in axon growth and in addition a striking impairment of granule neuron migration in the developing cerebellum. Individual knockdown of p250GAP and Smurf1 both revealed an overt short axon phenotype and no enhancement of this effect in the double knockdown suggesting that p250GAP and Smurf1 act synergistically in axon growth control. In migration however, the p250GAP/Smurf1 double knockdown results in an additive effect reflected by a more severe migration defect. This led us to conclude that they may function in parallel pathways to regulate migration. While Smurf1 and p250GAP may mostly target RhoA to control axon growth, the regulation of migration by Smurf1 and p250GAP may occur by targeting other substrates and Rho family members, respectively. Smurf1 has previously been identified as an E3 ligase for Par6 [6], and Par6 in turn is known to be involved in neuronal migration [38]. Also, in addition to RhoA, p250GAP has been demonstrated to act on Cdc42 [28], whose role in neuronal migration has been reported [39], [40]. Taken together our findings underscore the crucial role of RhoA regulators in the developing mammalian brain and the importance of the tight regulation of RhoA by the Cdh1-APC/p250GAP/Smurf1 pathway (**Figure 7**). A recent report suggests that the Cdh1 target SnoN also controls migration as SnoN knockdown affects the positioning in the internal granule layer of the cerebellum [41], indicating that Cdh1-APC governs several, possibly interconnected mechanisms of neural development.

## Materials and Methods

### Ethics Statement

All experiments involving live animals have been conducted according to the animal protocol approved by the “Verbraucherschutz und Lebensmittelsicherheit” of Lower Saxony, Germany (33.11.42502-04-058/08).

### Plasmids and Reagents

The Cdh1 RNAi, Smurf1 RNAi and Smurf1 DBM3/4 expression plasmids have been described previously [12], [16]. The p250GAP RNAi plasmid was generated by cloning a previously described p250GAP targeting region [29] into a modified pBluescript-U6 plasmid. Myc-p250GAP-N and Myc-p250GAP-C plasmids were generated by cloning fragments 1–2580 and 2581–5214, respectively into pCMVmyc plasmid. Myc-ubiquitin-p250GAP and Myc-p250GAP-ubiquitin fusion constructs were obtained by sub-cloning human ubiquitin from a pCMV5-HIS-ubiquitin plasmid into the pcDNA3-Myc-p250GAP plasmid.

### Primary Neuron Culture and Transfections

Cerebellar granule neurons were isolated from Wistar rats at postnatal day (P) 6 as described previously [42]. Neurons were plated on polyornithine-coated glass coverslips and kept in Basal Medium Eagle (BME) (Invitrogen, Carlsbad, CA) containing 10% calf serum (Hyclone Laboratories, Logan, UT), 25 mM KCl and 2 mM penicillin, streptomycin and glutamine (PSG). At P6+1 day *in vitro* (DIV), neurons were treated with 10 µM of the mitotic inhibitor cytosine β-D-arabinofuranoside to inhibit proliferation of non-neuronal cells. Granule neurons were transfected 8 h after plating ( = DIV 0) using the modified calcium phosphate method with indicated plasmids together with a GFP expression plasmid to visualize transfected cells and a plasmid encoding the anti-apoptotic protein Bcl-xl to rule out any effect of the genetic manipulations on cell survival. Expression of Bcl-xl itself has little or no effect on axon length [12]. For morphological assays, neurons on 12 mm cover slips were transfected with a total of 2 µg of plasmid together with 0.3 µg Bcl-xl and 0.2 µg GFP plasmids. The respective empty vectors served as control. The transfection ensures that at least 85% of the GFP-positive neurons co-express two other plasmids. Transfected neurons were fixed in 4% paraformaldehyde at DIV 4, permeabilized in 0.4% Triton-X100 in PBS and subjected to immunostaining using the rabbit GFP antibody (Invitrogen, Carlsbad, CA) followed by a fluorophore-conjugated anti-rabbit secondary antibody at 1∶1000 dilution in BME+10% horse serum.

### Axon Growth Assays

Morphological assays were performed as described previously [12] [17]. GFP-positive neurons were imaged by a blinded observer using a Nikon Eclipse TS100 (Tokyo, Japan) epifluorescence microscope and axonal lengths were measured using ImageJ software. Statistical tests (ANOVA or unpaired t-tests) were performed using Graph Pad Prism 5.0c software.

### Co-immunoprecipitation, Subcellular Fractionation, Ubiquitination Assays and Immunoblotting

For co-immunoprecipitation assays, transfected 293T cells (exogenous) or P9 mice cortices (endogenous) were lysed in buffer containing 150 mM NaCl, 210 mM Tris pH 7.4, 1 mM EDTA, 1% NP40, 10% glycerol and fresh protease inhibitors. Lysates were incubated with either c-Myc (Santa Cruz Biotechnology, CA), HA (Covance, Princeton, NJ), Cdh1, or GFP (Invitrogen) antibody at 4°C for 4 h in a tumbler and then with Protein A Sepharose (GE Healthcare) beads for 1 h. The beads were washed 3 times with Triton-X100 buffer (50 mM Tris-HCl pH7.5, 1 M NaCl, 1 mM EDTA, 1% triton-X100) and once with PBS. The beads were finally boiled in SDS-Laemmli buffer to elute the bound protein.

For subcellular fractionation, granule neurons in a 10 cm plate were scraped into detergent-free buffer A (10 mM HEPES pH7.9, 10 mM KCl, 0.1 mM EDTA, 0.1 mM EGTA, fresh protease inhibitors) and mechanically sheared using a 2 ml dounce homogenizer. Nuclei were spun down at 500 *g*, 4°C and the postnuclear supernatant fraction was transferred to a fresh tube. The nuclear pellet was washed twice in 0.1% NP40 supplemented buffer A and then lysed in buffer B (20 mM HEPES pH7.9, 400 mM NaCl, 1 mM EDTA, 1 mM EGTA, fresh protease inhibitors) and centrifuged at maximum speed at 4°C. The supernatant was collected as the nuclear fraction.

For cell-based ubiquitination assays, transfected 293T cells were lysed in a denatured modified RIPA buffer (50 mM Tris pH7.5, 150 mM NaCl, 1% NP-40, 0.1% SDS, 0.5% sodium deoxycholate, 10 mM N-ethyl maleimide and fresh protease inhibitors) to reduce detection of non-specific ubiquitination, as described previously [43]. The lysates were incubated with the Myc antibody at 4°C for 2 h and then with Protein A Sepharose beads for 45 min. The beads were washed 3 times with triton-X100 buffer to remove any unbound protein and once with PBS. The bound protein was eluted by boiling the beads in SDS-Laemmli buffer. SDS-PAGE and Western blotting were performed as previously described [18].

### In Vivo Electroporation

In vivo electroporation was performed as previously described [17]. Plasmid DNA in PBS (4 µl/animal) together with 0.3% fast green was injected into the cerebellar cortex of P4 rat pups using a 25 µl syringe (Hamilton, Bonaduz, Switzerland) with a 30-guage needle. 16 µg of the U6-CMV-EGFP or p250GAP RNAi/Smurf1 RNAi-CMV-EGFP bicistronic plasmid and 4 µg Bcl-x_L_ expression plasmid were injected per animal. Following DNA injection, animals were subjected to repeated electric pulses (5 pulses of 155–170 V for 50 ms with intervals of 950 ms) and allowed to recover on a heating pad. Electroporated pups were returned to the mother until P9. Coronal sections (40 µm) of cerebella were subjected to immunohistochemistry using a mouse GFP antibody (Santa Cruz). Approximately 90% of GFP-positive granule neurons were associated with parallel fibers ( = parallel fiber index) in the control condition [17].

### Confocal Imaging

For confocal imaging of granule neurons transfected with GFP-p250GAP, we used a Leica TCS SP2 confocal microscope with a 63x oil objective.

## Supporting Information

Figure S1 **RhoGAP Screen. A.** 293T cells were transfected with control vector pEGFP and HA-Graf, pTB701 and GFP-Cdh1 or HA-Graf and GFP-Cdh1 plasmids and lysates were subjected to immunoprecipitation with the HA antibody followed by immunoblotting with GFP antibody. **B.** 293T cells were transfected with control vector pEGFP and HA-Cdh1, pCMV5 and GFP-Nadrin or HA-Cdh1 and GFP-Nadrin plasmids and lysates were subjected to immunoprecipitation with the HA antibody followed by immunoblotting with GFP antibody. **C.** 293T cells were transfected with control vector pcDNA3.1 and HA-Cdh1, pCMV5 and Myc-Ophn1 or HA-Cdh1 and Myc-Ophn1 plasmids and lysates were subjected to immunoprecipitation with the HA antibody followed by immunoblotting with Myc antibody. **D.** 293T cells were transfected with control vector pKH3 and GFP-Cdh1, pEGFP and HA-p190GAP or GFP-Cdh1 and HA-p190GAP plasmids and the lysates were subjected to immunoprecipitation with the GFP antibody followed by immunoblotting with the HA antibody. **E.** 293T cells were transfected with control vector pcDNA3 and HA-Cdh1, pCMV5 and Myc-p250GAP or HA-Cdh1 and Myc-p250GAP plasmids and lysates were subjected to immunoprecipitation with the HA antibody followed by immunoblotting with Myc antibody.
(TIF)

Figure S2 **Cdh1 interacts with p250GAP D-box mutant.** Lysates of 293T cells transfected with GFP-Cdh1 plasmid together with control vector pcDNA3, Myc-p250GAP wild-type (Myc-p250GAP WT) or the putative D-box mutant (Myc-p250GAP DBM) were subjected to immunoprecipitation using the Myc antibody followed by immunoblotting with the GFP antibody.
(TIF)

Figure S3 **Quantification of processes in the developing cerebellar cortex. A.** Quantification of thin (normal) processes and short, thick processes in control, p250GAP knockdown and p250GAP/Smurf1 double-knockdown conditions. A total of 1200 processes were counted (ANOVA, ***p<0.0001, values indicate mean+SEM).
(TIF)
