# Supplementary figures and images for: p250GAP Is a Novel Player in the Cdh1-APC/Smurf1 Pathway of Axon Growth Regulation
Source: PLoS One. 2012 Nov 30;7(11):e50735. doi: 10.1371/journal.pone.0050735 (PMC3511349; doi:10.1371/journal.pone.0050735)

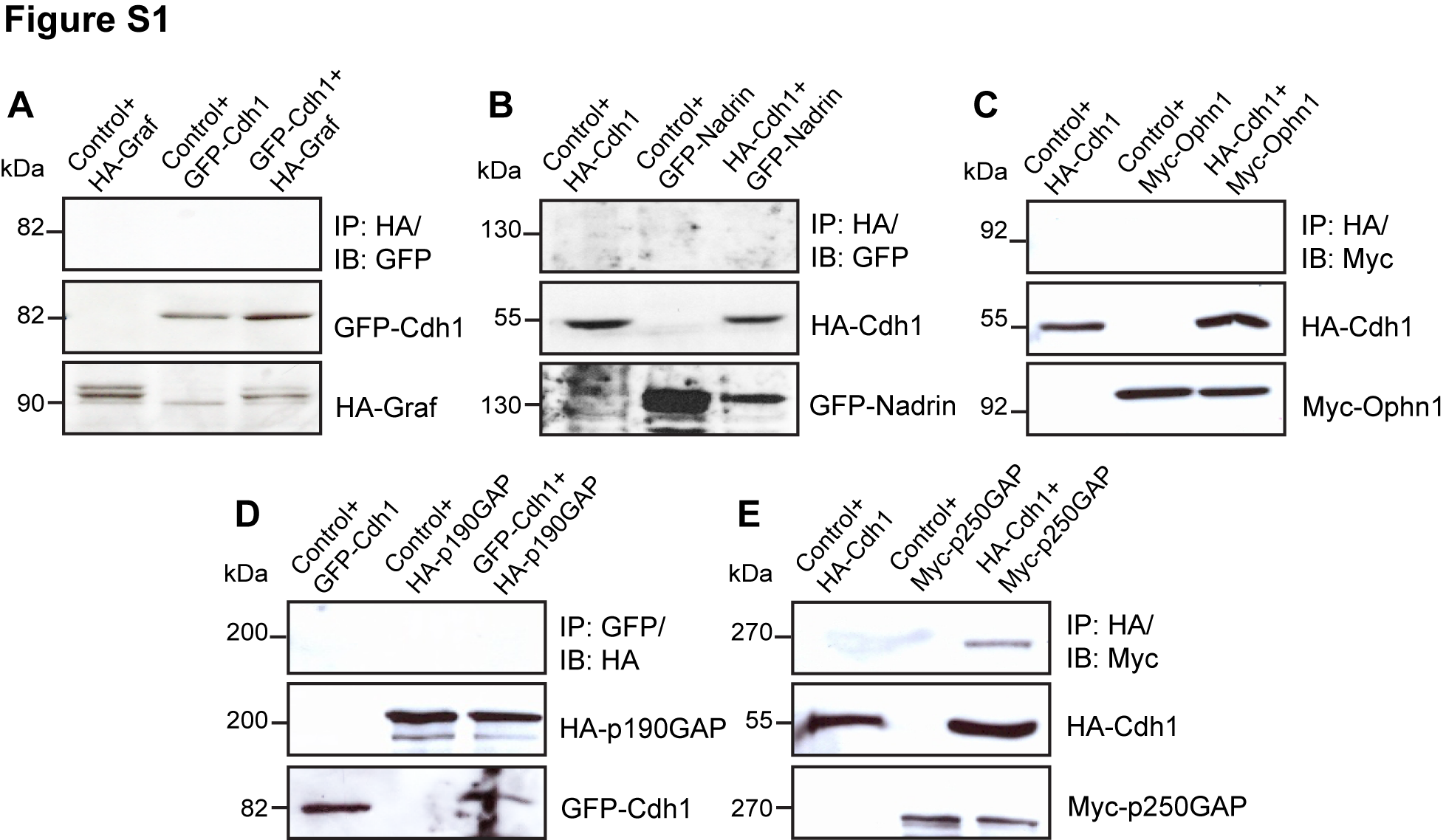

Supplement: Figure S1 — RhoGAP Screen. A. 293T cells were transfected with control vector pEGFP and HA-Graf, pTB701 and GFP-Cdh1 or HA-Graf and GFP-Cdh1 plasmids and lysates were subjected to immunoprecipitation with the HA antibody followed by immunoblotting with GFP antibody. B. 293T cells were transfected with control vector pEGFP and HA-Cdh1, pCMV5 and GFP-Nadrin or HA-Cdh1 and GFP-Nadrin plasmids and lysates were subjected to immunoprecipitation with the HA antibody followed by immunoblotting with GFP antibody. C. 293T cells were transfected with control vector pcDNA3.1 and HA-Cdh1, pCMV5 and Myc-Ophn1 or HA-Cdh1 and Myc-Ophn1 plasmids and lysates were subjected to immunoprecipitation with the HA antibody followed by immunoblotting with Myc antibody. D. 293T cells were transfected with control vector pKH3 and GFP-Cdh1, pEGFP and HA-p190GAP or GFP-Cdh1 and HA-p190GAP plasmids and the lysates were subjected to immunoprecipitation with the GFP antibody followed by immunoblotting with the HA antibody. E. 293T cells were transfected with control vector pcDNA3 and HA-Cdh1, pCMV5 and Myc-p250GAP or HA-Cdh1 and Myc-p250GAP plasmids and lysates were subjected to immunoprecipitation with the HA antibody followed by immunoblotting with Myc antibody. (TIF) [file pone.0050735.s001.tif]

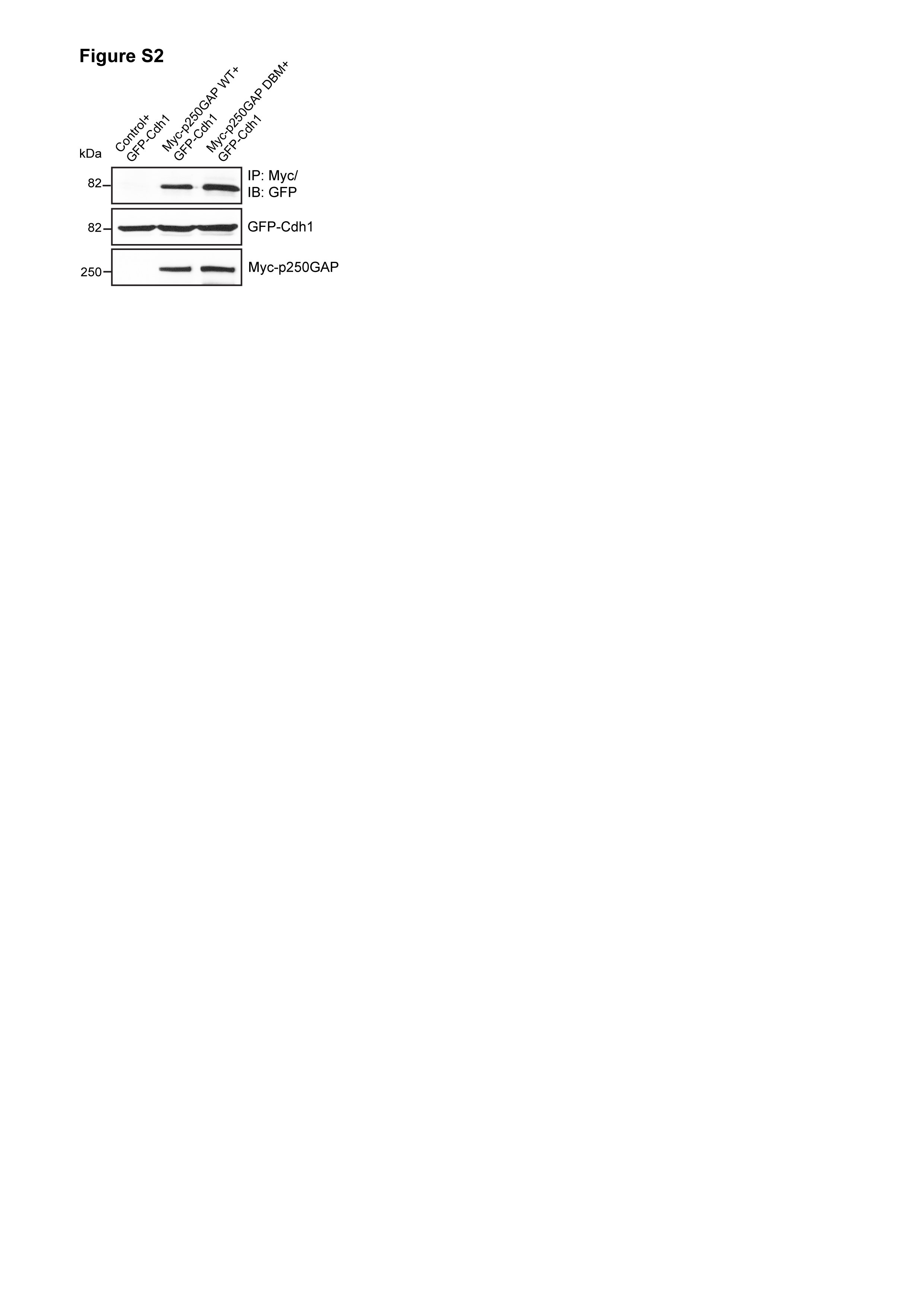

Supplement: Figure S2 — Cdh1 interacts with p250GAP D-box mutant. Lysates of 293T cells transfected with GFP-Cdh1 plasmid together with control vector pcDNA3, Myc-p250GAP wild-type (Myc-p250GAP WT) or the putative D-box mutant (Myc-p250GAP DBM) were subjected to immunoprecipitation using the Myc antibody followed by immunoblotting with the GFP antibody. (TIF) [file pone.0050735.s002.tif]

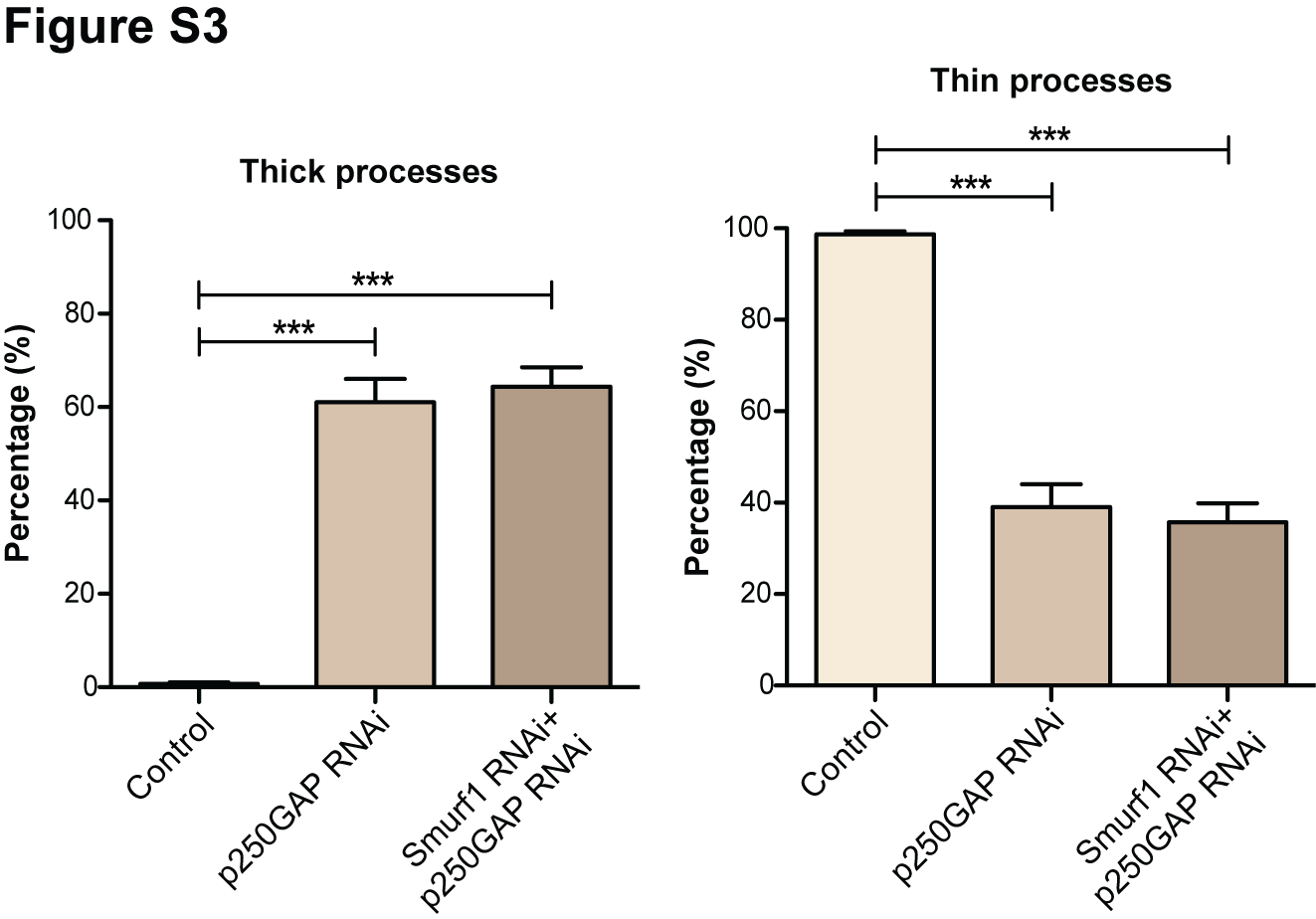

Supplement: Figure S3 — Quantification of processes in the developing cerebellar cortex. A. Quantification of thin (normal) processes and short, thick processes in control, p250GAP knockdown and p250GAP/Smurf1 double-knockdown conditions. A total of 1200 processes were counted (ANOVA, ***p<0.0001, values indicate mean+SEM). (TIF) [file pone.0050735.s003.tif]
